# Pigmented lesions with unusual penile localisation: Usefulness of reflectance confocal microscopy ‐ Report of 2 cases

**DOI:** 10.1111/ajd.13615

**Published:** 2021-05-10

**Authors:** Tea Rocco, Alessandra Ventura, Valeria Ciciarelli, Maria Concetta Fargnoli

**Affiliations:** ^1^ Dermatology Department of Biotechnological and Applied Clinical Science University of L’Aquila L’Aquila Italy

## INTRODUCTION

Pigmented skin lesions of the penis include melanocytic and non‐melanocytic lesions.^1^ Melanocytic lesions on the penis occur in 10–12% of the general population with melanoma accounting for 1.4% of all penile cancers and less than 0.1% of melanoma cases.^2^

Reed nevus is a deep pigmented melanocytic lesion, commonly located on the lower extremities of young adults and the penile location of this lesion is rarely reported^3^. The starbust pattern is typical of Reed nevus but it has been also reported in melanoma. Pigmented Bowen disease is an *in situ* squamous cell carcinoma, clinically appearing as a slow‐growing, well‐defined, hyperpigmented plaque and its location on the genital area is uncommon in white populations.^4^

Reflectance confocal microscopy (RCM) is a non‐invasive diagnostic technique that improves the diagnostic accuracy of equivocal pigmented and non‐pigmented skin lesions. Only few case reports described RCM features of pigmented genital lesions.^5^

Herein, we report two common pigmented lesions with an unusual penile localisation in which RCM has been useful for diagnosis and management.

## CASE 1

**Figure 1:**
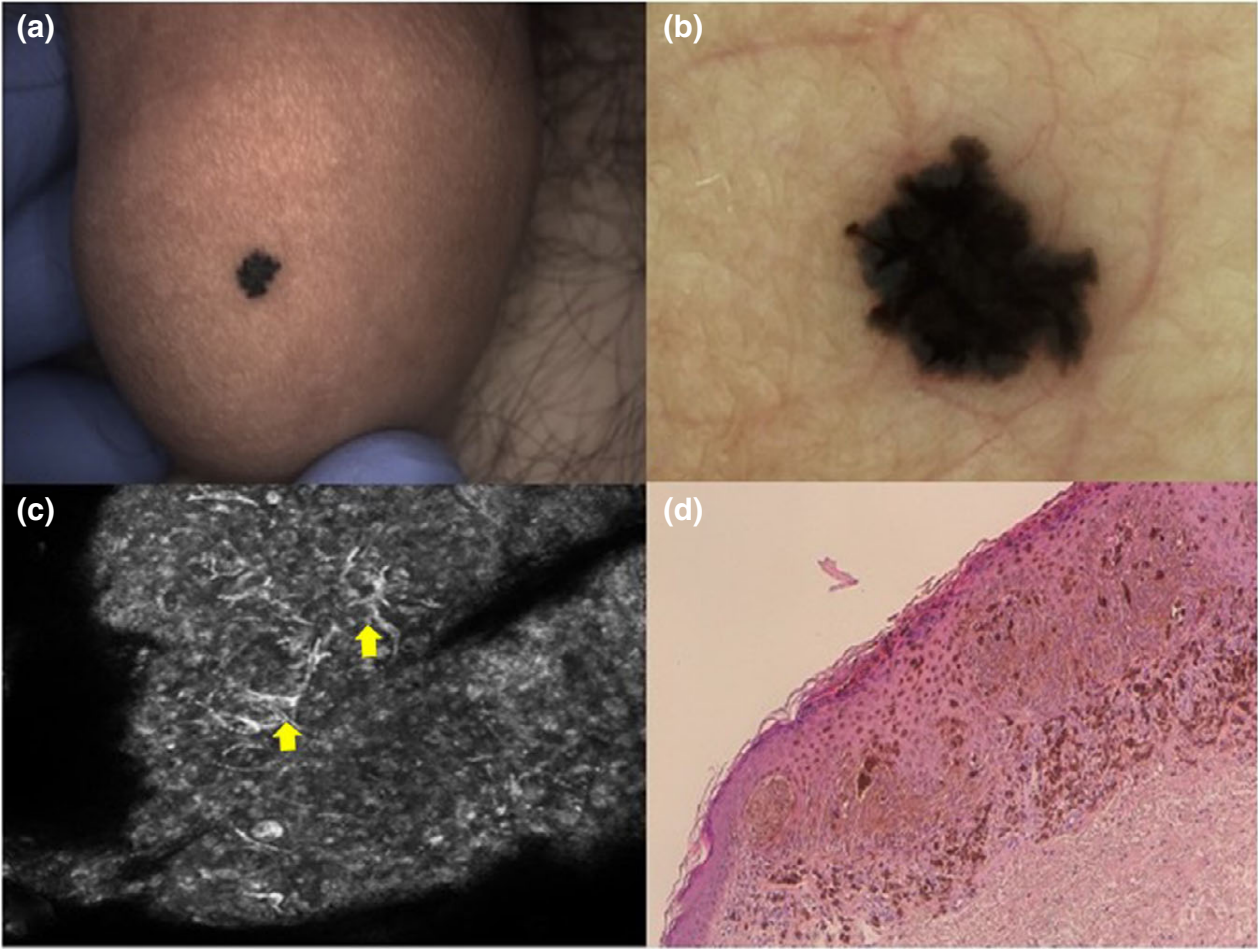
(a) Clinical image: brown/black irregular macule on the glans penis. (b) Dermoscopy shows a starbust pattern with a central blotch of black/dark brown colour and some asymmetric pseudopods at the periphery. (c) RCM image with spindle cells (yellow arrows). Single image = 400 × 500 µm. (d) Histopathologic features of Reed nevus with typical spindled melanocytes in interconnected fascicles (H&E staining. 20x magnification). RCM, Reflectance Confocal Microscopy.

A 16‐year‐old boy presented with a 4 mm brown/black, irregularly shaped macule on the glans penis, that appeared 3 months earlier and changed over time (Fig. 1a). Dermatoscopic examination showed an irregular starbust pattern suggestive of Reed nevus (Fig. 1b). Due to the unusual penile location and differential diagnosis with melanoma, we performed RCM that revealed multiple and diffuse spindle cells in the epidermis with sharp lateral demarcation (Fig. 1c). These features were considered to be characteristic of Spitz nevi and supported the diagnosis which was histopathologically confirmed after excisional biopsy (Fig. 1d).

## CASE 2

**Figure 2:**
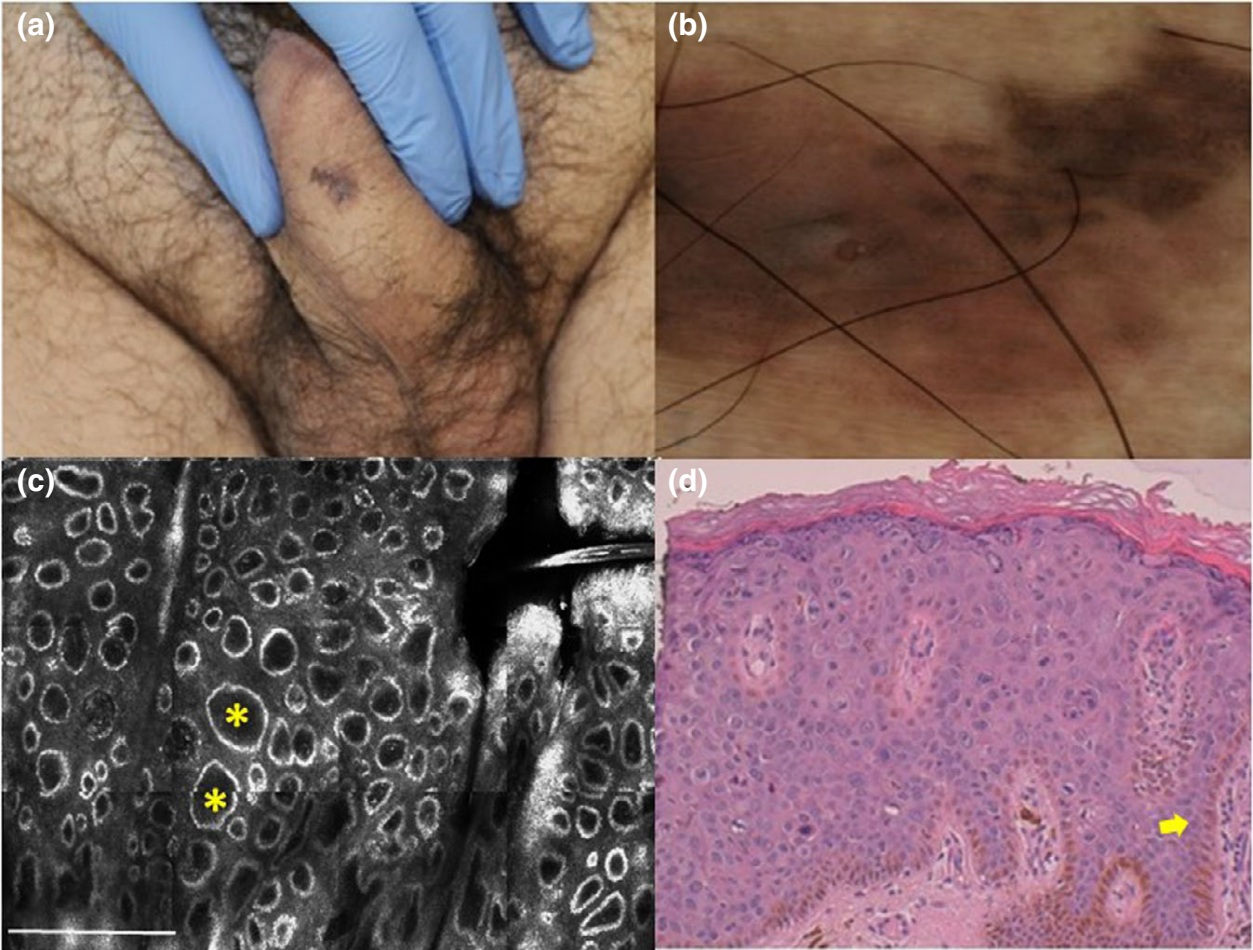
(a) Clinical image: pink/brown macule on the penis shaft. (b) Dermatoscopy (x30) shows an eccentric hyperpigmented area and irregularly distributed brown/black globules on a pinkish background (c) High magnification RCM examination at DEJ with edged dermal papillae as bright rings (yellow asterisks). White bar = 500 µm. (d) Histopathology of pigmented Bowen disease with acanthotic epidermis, thickening of rete ridges and basal pigmented keratinocytes (yellow arrow) (H&E staining. 10x magnification). RCM, Reflectance Confocal Microscopy.

A 45‐year‐old man presented with a 1 cm asymptomatic pink/brown macule on the penis shaft that had appeared one year earlier (Fig. 2a). Dermatoscopy showed non‐specific features with a hyperpigmented area and irregularly distributed brown/black globules on a pinkish background (Fig. 2b). RCM showed an atypical honeycomb pattern and edged dermal papillae appearing as bright rings in the absence of junctional and dermal nests of melanocytic cells (Fig. 2c). The cytological atypia of keratinocytes and the absence of diagnostic confocal features of melanocytic lesion supported the diagnosis of keratinocyte carcinoma. Histopathological examination of an incisional biopsy confirmed the diagnosis of pigmented Bowen disease (Fig. 2d).

## DISCUSSION

The diagnosis of clinically atypical pigmented lesions on the genitalia can be challenging since differential diagnosis with melanoma might be difficult.

RCM has been recently shown to play a role in the non‐invasive diagnosis of vulvar nevi,^6^ providing additional information to the clinical and dermatoscopic examination. In spitzoid lesions, it is not always possible to differentiate a nevus from a melanoma because it is impossible to explore the lesion in significant vertical depth. However, the presence of sharp border cut‐offs and spindle cells, as in our case, is more characteristic of a nevus. In our patients, RCM has provided a near‐histological morphological accuracy that helped us in the diagnosis of Reed nevus and keratinocyte carcinoma, respectively.

Genital disorders are generally a source of anxiety and a biopsy in this sensitive area is not preferred for the related pain and risk of scarring thus a fast and non‐invasive diagnosis is highly desirable. In addition, RCM reduces the risk of infection as well as costs, resources and time.

In conclusion, RCM is an additional tool that can be used in clinical practice to aid clinical diagnosis of pigmented lesions, especially if located in sensitive areas such as genitalia where unnecessary surgery is preferably avoided.
